# Early malfunction of a biliary self-expandable metal stent with an antireflux valve: A case report

**DOI:** 10.1097/MD.0000000000019750

**Published:** 2020-04-17

**Authors:** Sang Hoon Kim, Chi Hyuk Oh, Min Lee Jae, Seong Ji Choi, Hyuk Soon Choi, Eun Sun Kim, Bora Keum, Yoon Tae Jeen, Hoon Jai Chun, Hong Sik Lee, Chang Duck Kim

**Affiliations:** a Division of Gastroenterology and Hepatology, Department of Internal Medicine, Korea University College of Medicine; b Division of Gastroenterology and Hepatology, Department of Internal Medicine, Kyung Hee University College of Medicine, Seoul, Korea.

**Keywords:** antireflux valve, bile duct obstruction, expanded polytetrafluoroethylene, malfunction, self-expandable metal stent

## Abstract

**Rationale:**

Biliary drainage is essential to resolve jaundice in patients with malignant biliary obstruction. Recently, a biliary self-expandable metal stent (SEMS) with an antireflux valve was developed to prevent enteric-biliary reflux. Its antireflux valve was made of expanded polytetrafluoroethylene (ePTFE), a biostable and biocompatible material. Changes of the ePTFE membrane of medical devices are rarely reported in clinical practice.

**Patient concerns:**

A 59-year-old woman was admitted with a complaint of jaundice. Around 1 month before, she underwent endoscopic biliary stenting using a SEMS with an antireflux valve.

**Diagnosis:**

Acute cholangitis due to stent clogging was detected on abdominal computed tomography.

**Interventions:**

Endoscopic retrograde cholangiopancreatography and removal of the SEMS with deformity were performed.

**Outcomes:**

The obstructive jaundice was rapidly resolved after the removal of the clogged stent.

**Lessons:**

Although the ePTFE membrane is widely used to make medical devices, unexpected changes could occur after the membrane is exposed to bile juice in the duodenum. When performing biliary stenting using a SEMS with an antireflux valve, both the risk of early malfunction and the benefit of the antireflux effect should be considered.

## Abstract 2

Supplemental Digital Content is available in the text

## 1 Introduction

Biliary stenting is one of the therapeutic procedures for resolving of biliary obstruction in patients with pancreaticobiliary tumors. The endoscopic approach can be used for biliary stenting to avoid an invasive procedure such as the percutaneous approach. Moreover, a self-expandable metal stent (SEMS) has better endurance and stability than a plastic stent and has been widely used for palliation of malignant biliary obstruction.^[1–5]^ However, enteric-biliary reflux led to biliary sludge, and food impaction easily induced cholangitis due to the widened ampulla after stenting. It is known as a major cause of SEMS occlusions.^[6]^ Recently, a modified SEMS with an antireflux valve was developed. It had an uncovered proximal end and Winsock-shaped duodenal end made of expanded polytetrafluoroethylene (ePTFE).^[7–12]^ The ePTFE membrane, which is widely used as a vascular graft, stent cover, and cardiovascular patch, is characterized by chemical stability, a hydrophilic surface, excellent flexibility, and good biocompatibility. In previous studies, ePTFE was shown to be stronger, tougher, and more stable than other materials such as silicone.^[13–15]^ In addition, the modified SEMS with an antireflux valve had better clinical outcomes.^[11]^ The stent is considered to have both long durability and antireflux effect. However, despite the positive results of many experimental studies, unexpected reaction or changes of the medical device may be occur in clinical practice.

Here, we present a case of early biliary stent occlusion induced by a hardened antireflux valve made of ePTFE.

## 2 Case report

**Figure 1:**
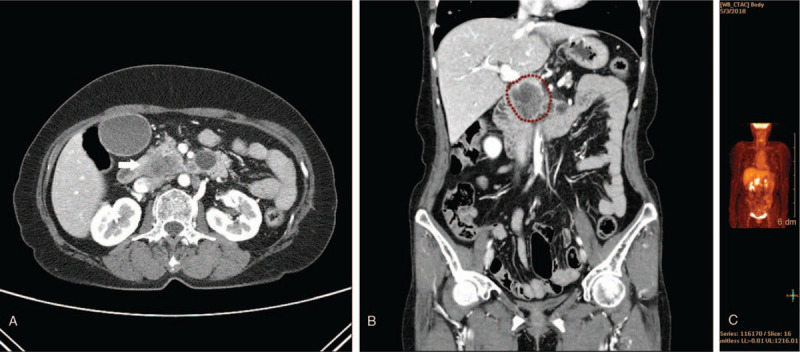
(A) Enhanced computed tomography (CT) reveals mass of uncinate process of pancreas (white arrow) with regional enlarged lymph nodes in portocaval area, porta hepatis area. Biliary tree dilation with abrupt narrowing beside the mass suggests distal common bile duct invasion. (B) A 4.2-cm-sized mass (dotted measuring line) extending vertically to portocaval area was noted. (C) Hypermetabolic lesions in both lung fields, pancreatic head, lymph nodes of porta hepatis, left paraaortic area, and sigmoid colon in positron emission tomography-CT.

A 59-year-old female patient was admitted because of jaundice and general weakness. On the computed tomography (CT) scan, a pancreatic head mass with lung nodules was detected (Fig. 1A and B). The initial laboratory findings were as follows: aspartate aminotransferase level, 275 IU/L; alanine aminotransferase level, 413 IU/L; total bilirubin level, 14.9 mg/dL; and direct bilirubin level, 8.7 mg/dL. The mass was confirmed to be pancreatic adenocarcinoma by endoscopic ultrasonographic fine-needle aspiration. Multiple hypermetabolic lesions were found not only in the pancreatic head but also in the lung, pelvic cavity, and peripancreatic area on positron emission tomography-CT (Fig. 1C). Biliary stenting was performed using a metal stent (uncovered metal stent, 7 cm; Mitech Co Ltd, Seoul, South Korea) into the bile duct. After metal stenting, the total bilirubin level decreased to <3.0 mg/dL. As her Eastern Cooperative Oncology Group performance status score was 1 to 2, 1st-line chemotherapy was initiated with gemcitabine and nab-paclitaxel. Despite the systemic chemotherapy for 4 months, cancer progression was observed on the follow-up CT scan, and the therapeutic plan was changed to conservative treatment.

**Figure 2:**
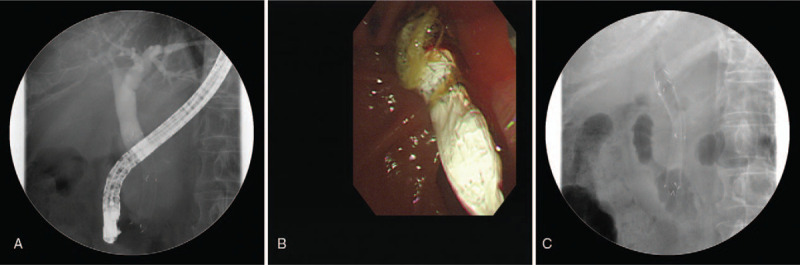
(A) A fluoroscopic image after removing clogged inner full-covered metal stent. (B) After the removal of previous inner metal stent, antireflux valve stent was inserted. (C) A fluoroscopic mage of an external uncovered metal stent with an internal antireflux valve stent.

During the course of the chemotherapy, she had repeated aggravation of jaundice due to food impaction and stent clogging. Therefore, an antireflux valve stent (8 cm; S&G BioTech Inc, Seoul, South Korea) was inserted after removal of the inner full-covered metal stent. Finally, an external uncovered metal stent with an internal antireflux valve stent was inserted (Fig. 2).

**Figure 3:**
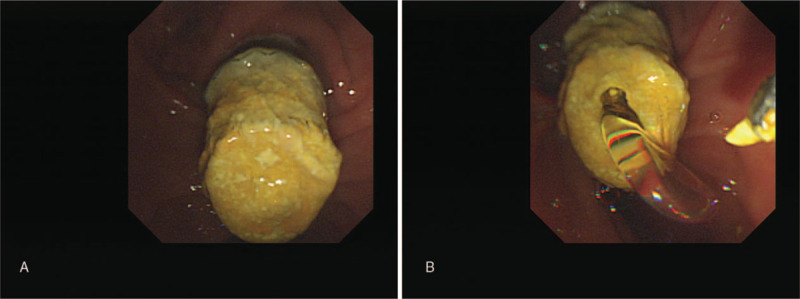
(A) A stiffened stent liner clogged by sludge was observed. Anti-reflux valve is tightly obstructed. (B) A forcep was used to make a hole for bile juice drainage.

She was admitted because of severe abdominal pain and septic condition 1 month after insertion of an antireflux valve stent. A diffuse dilated peripheral bile duct was observed, but no filling defect was found in the common bile duct and metal stent. In the endoscopic view of duodenoscopy, a hardened and tightly stuck antireflux valve was found (Fig. 3).

**Figure 4:**
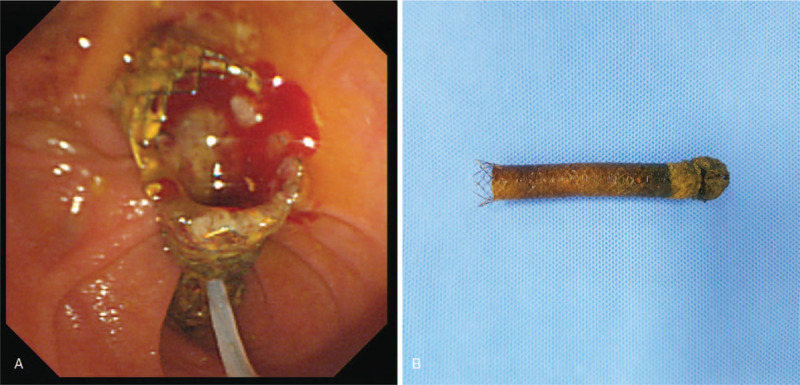
(A) By using snare basket, the torn and fragmented inner antireflux metal stent was endoscopically removed. (B) Image of the extracted antireflux metal stent.

The flexible liner tip was firmly obstructing the reflux tip with deformity, making intraluminal cannulation through the stent impossible. Therefore, forceps were used to roughly tear the stent, and bile juice gushed out after partial tearing of the hardened portion of the stent (see Supplemental Video 1; Forceps were used to roughly tear the hardened stent opening, and congested bile juice gushed out afterwards). Then, the antireflux valve stent was extracted using a snare basket (Fig. 4). Instead of another metal stent, 2 double pigtail stents were inserted through the firstly inserted metal stent to prevent food impaction.

Six weeks later, a partially covered metal stent (9 cm; BONASTENT, Standard Sci Tech Inc, Seoul, South Korea) replaced the plastic pigtail stents after confirming these stents being clogged by endoscopic retrograde cholangiography. Hyperbilirubimenia was improved (total bilirubin 8.96 → 7.53) after the change of stents. Unfortunately, the patient died 1 week later due to acute respiratory distress syndrome.

Informed consent was obtained from the patient for the purpose of publication.

## 3 Discussion

The ePTFE is a smooth and relatively stable material suitable for use in humans. It is widely used as a biomaterial because it is not easily deformed in either acidic or basic conditions and is highly permeable for gas. An antireflux SEMS, which has a long liner with ePTFE, is a recently developed stent that has been reported to have benefits for antireflux and maintaining stent patency.^[16,17]^ However, the 1st clinical case of using an ePTFE-based antireflux SEMS, which induced cholangitis because of a solid obstruction of the reflux valve area, is exceptional.

**Figure 5:**
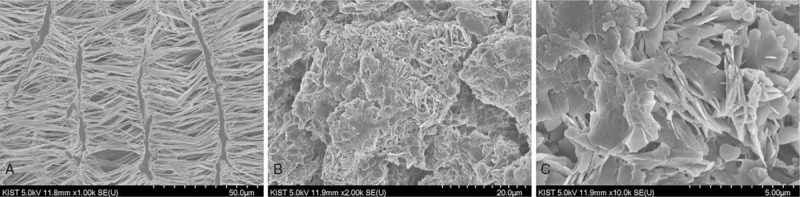
Electron microscopic images of the antireflux liner area made of expanded polytetrafluoroethylene (ePTFE). (A) Normal e-PTFE membrane before use. (B) Stiffened ePTFE membrane at low magnification. (C) Stiffened ePTFE membrane at high magnification.

Biliary stent insertion using endoscopic retrograde cholangiopancreatography is an effective treatment for biliary tract obstruction. However, transpapillary SEMS placement is associated with problems such as food regurgitation from the duodenum to the open biliary tract^[18–20]^ and sphincter of Oddi dysfunction.^[21]^ In this case, an antireflux stent was inserted in the previous stent because of recurrent food regurgitation to the bile duct. However, the cholangitis recurred 1 month after owing to the stiffening of the antireflux valve. Figure 5 demonstrates an electron microscopic image of the antireflux liner area made of ePTFE. Figure 5A is a normal ePTFE membrane before use, and Figure 5B and C shows the stiffened ePTFE membrane at low and high magnifications.

The normal ePTFE membrane has a fiber-form surface; however, the stiffened ePTFE membrane lost its form because of environmental exposure to bile juice. Despite the relative stability of ePTFE stents even when exposed to basic liquids and bile juice in experimental settings (W. L. Gore & Associates, unpublished data, 2000), unexpected reactions may still occur when the stent is exposed to human biliary environment. The possible causes of the unexpected stiffening are friction by contact between the 2 stents in a stent-in-stent manner, a reaction to the contact between the surfaces of the 2 different materials, and interaction between the stent and certain bile juice components.

Stents made of ePTFE with an antireflux valve are effective in preventing regurgitations; however, when inserting the stent in the lumen of another covered biliary stent with a different membrane material in the stent-in-stent manner, short-term stiffening of the valve should be considered. Similarly, a recent multicenter randomized controlled trial in Japan insisted that use of an antireflux metal stent for nonresectable distal malignant biliary obstruction was not associated with longer time to recurrent biliary obstruction compared to the covered SEMS.^[22]^ Our case also supports the necessity for improvements of the antireflux valve and more effective strategies to prevent ePTFE stiffening.

In the future, we hope to understand the causes of the stiffening through an analysis of shared properties of multiple case reports and to reach a consensus for improving the efficacy and safety of stents made of ePTFE.

## Author contributions

**Data curation:** Sang Hoon Kim, Chi Hyuk Oh.

**Investigation:** Sang Hoon Kim, Chi Hyuk Oh.

**Writing – original draft:** Sang Hoon Kim, Chi Hyuk Oh, Jae Min Lee.

**Writing – review & editing:** Jae Min Lee, Seong Ji Choi, Hyuk Soon Choi, Eun Sun Kim, Bora Keum, Yoon Tae Jeen, Hoon Jai Chun, Hong Sik Lee, Chang Duck Kim.

Jae Min Lee orcid: 0000-0001-9553-5101.

## Supplementary Material

Supplemental Digital Content
